# Research on the diffusion plugging mechanism of flowing water grouting slurry in karst pipelines

**DOI:** 10.1038/s41598-024-65852-1

**Published:** 2024-08-20

**Authors:** Shuai Liu, Bo Peng, Jie Liu, Ming yuan Wang, Gang Li

**Affiliations:** 1 Key Laboratory of Geological Hazards On Three Gorges Reservoir Area, Ministry of Education, China Three Gorges University, Yichang, 443002 China; 2 College of Civil Engineering and Architecture, China Three Gorges University, Yichang, 443002 China; 3 School of Civil Engineering and Architecture, China Jishou University, Zhangjiajie, 427000 China

**Keywords:** Karst pipeline, Grouting sealing, Simulation test, Plugging mechanism, Civil engineering, Composites

## Abstract

Among the many adverse geological disasters, the surge water disaster in karst areas causes the greatest loss to underground engineering construction, so it is necessary to carry out relevant research on the management of surge water disaster in karst pipelines. This study presents the creation of an oily epoxy resin magnetic convergence grouting material (OEMS) specifically developed to prevent water infiltration in pipelines. A self-designed visual karst pipeline grouting simulation system was used to conduct an experimental study on the diffusion and plugging behavior of magnetic slurry grouting. A model was constructed to simulate the migration of a magnetic slurry in water inrush circumstances. The model is based on the theory of slurry diffusion and the concept of magnetic adsorption. The results suggest that:(i) The best performance in grouting sealing is achieved when the ratio of new OEMS epoxy resin A liquid to B liquid is 2:1, and the blending ratio of flyash and Fe_3_O_4_ powder falls between 25 and 55%. (ii) The primary and secondary correlations among the parameters that affect the rate of change in flow rate, plugging pressure, and slurry retention rate are as follows: Hydrodynamic velocity has the greatest correlation, followed by plugging length, Fe_3_O_4_ power ratio, and flyash mixture ratio. (iii) The validity of the model is verified by comparing empirical observations with calculated theoretical values.

## Introduction

Due to China’s fast-growing economy, the size and quantity of underground engineering projects, such as tunnels, are expanding. China, being the country with the largest and most widespread karst formations in the world, is highly susceptible to geological disasters such as water inrush, mud inrush, and collapse during construction. These hazards pose a significant threat to project development^1–5^. Karst water inrush disasters have been responsible for the most significant economic losses and mortality in numerous disastrous geological events^6–8^. Hence, conducting pertinent study on the management of karst pipeline water influx calamity is of utmost significance.

The various geological features in the karst area give rise to varied disaster mechanisms, resulting in distinct forms of water inrush. The many types of water influx are categorized based on the water storage conditions and the characteristics of the buildings that cause disasters. These include fissure water influx, fault water influx, karst cave water influx, pipeline water influx, and subterranean river water influx^9^. Currently, grouting is a highly successful method for achieving water inrush prevention^10–13^. Currently, the liquidity of the typical cement slurry used in engineering is uncontrollable and pumpable period is not adjustable. And it has a high solubility in water and low resistance to erosion from flowing water washout .These characteristics make it unsuitable for meeting the requirements of engineering construction. Despite their high early strength and outstanding grout ability, chemical grouting materials are costly and pose possible environmental risks^14,15^.Water-soluble polyurethane and epoxy resins will affect their sealing effect under the action of dynamic water scour, and there are few studies at present. Furthermore, numerous studies have demonstrated that polymer cement-based modified composite materials enhance the cement's ability to resist dispersion to some degree. These materials are also resistant to erosion, and the grouting consolidation body benefits from corrosion resistance, freeze–thaw resistance, and impermeability, among other advantages^16^. Nevertheless, in karst regions, the current grouting materials are unable to effectively seal the high-pressure water flow. Hence, it is necessary to devise a novel grouting substance for the purpose of effectively sealing karst formation water inrush in projects, which holds immense importance.

The current body of research on karst water inrush grouting mostly centers around the dispersion of slurry within rock fissures under dynamic water conditions. Zhang et al.^17^ discovered the impact of grouting pressure, injection rate, and crack width on the effectiveness of fracture grouting by examining the rheology of C-S slurry. Chen et al.^18^ using a combination of finite element method and fluid volume technology to investigate a numerical model for simulating the crack grouting process in soil.

Based on the theoretical method of grouting design, Rafi and Stille^19^ studied the applicability of GIN method. Sui et al.^20^ studied the sealing effect of chemical grouting of rock fissures under dynamic water conditions through experiments. Wang et al.^21^ analyzed the hydraulic-mechanical interaction to examine the features of leakage and pipeline occurrence during the excavation of a channel tunnel. Wang et al.^22^ introduced a probabilistic evaluation approach that combines the hydraulic-mechanical coupling method with stochastic finite member analysis. The suggested approach allows for the appropriate consideration and analysis of the spatial variability of soil parameters in conjunction with dam situations. The results demonstrate that the suggested framework offers a graphical representation of the pipe flushing process and indicates that pipe erosion takes place in around 40% of the hydraulic samples. Xiao et al.^23^ developed a basic grouting model to forecast the diffusion pattern of grout in fracture channels. Liang et al.^4^ investigated the dispersion of chemical grouting in water flow and sand flow using an experimental grouting equipment designed for inclined fissures. Currently, the research findings on grouting theory mostly consist of seepage grouting theory^24–29^, fracture grouting theory^30,31^, and compaction grouting theory^32–34^. These theories are all founded on the principles of laminar flow and Darcy’s law. Nevertheless, karst pipeline-type water inrush exhibits the traits of substantial volume, elevated flow velocity, and turbulent dynamics. The conventional approach of using grouting to plug water is not appropriate for dynamic water pipelines, and it has a low slurry retention rate, which fails to effectively address the issue of water infiltration in karst pipeline-type disasters. Hence, it is imperative to innovate new plugging technology.

Magnetic fluid is a stable mixture created by dispersing nano-sized magnetic particles with surfactants in a base fluid^35,36^. The magnetic fluid has exceptional performance and finds applications in magnetic fluid sealing, shock absorption, medical equipment, and other industries that involve challenging conditions. However, its use in the engineering sector remains limited. Introducing steel fiber into concrete can significantly enhance its tensile strength, flexural strength, and compressive strength^37–41^. Liew et al.^42^ introduced a novel technique that utilizes magnetic fields to align steel fibers with the tensile stress or vertical crack plane of concrete structures. This technology efficiently reduces the energy consumption resulting from the random arrangement of fibers. In their study, Hajforoush et al.^43^ observed that the addition of 1.5% steel fiber to concrete resulted in an 18% increase in compressive strength and a 16% increase in flexural strength when subjected to a uniform magnetic field. Chen et al.^44^ investigated the bending and displacement of steel slag by substituting concrete aggregate with steel slag and subjecting it to an external magnetic field. The findings indicate that steel slag exerts a vibrational impact on concrete.

This work presents a composite grouting material that has been enhanced with Fe_3_O_4_ powder, based on the concept of the magnetorheological effect. The magnetic field produced by the magnetic rod is utilized to induce a magnetic convergence effect in the slurry, so enhancing its resistance to erosion and shear forces. This study presents the development of an OEMS specifically designed for grouting and sealing pipeline water inrush. The diffusion and plugging mechanism of dynamic underwater grouting slurry is investigated using a self-developed visual water inrush grouting simulation test system. Simultaneously, a model is developed to study the behavior of magnetic slurry in a magnetic field when water influx occurs. The computed values of the model are compared with the experimental test results to validate the soundness of the model. The research findings can offer direction for future technical applications.

## Experimental study of OMES

### Experimental material

**Figure 1:**
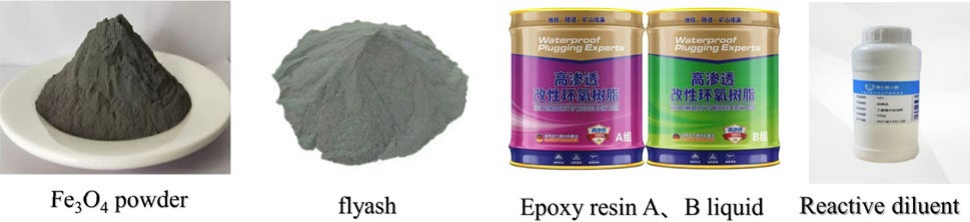
Materials used in the test.

The primary constituents utilized in the manufacturing process of OEMS consist of Fe_3_O_4_ powder, epoxy resin A, B liquid, flyash, and active diluent, as depicted in Fig. 1.

### Test scheme design

The team’s extensive scientific research experience and literature review have led to the selection of epoxy resin as the primary material for repairing wading buildings. Epoxy resin is chosen for its ability to facilitate the controlled flow of the slurry, its strong resistance to erosion, its effective water plugging and anti-permeability properties, and its ability to prevent dispersion. Additionally, the use of magnets helps to orient the attraction of the slurry to metal surfaces, enabling magnetic self-polymerization and guided flow.

**Figure 2:**
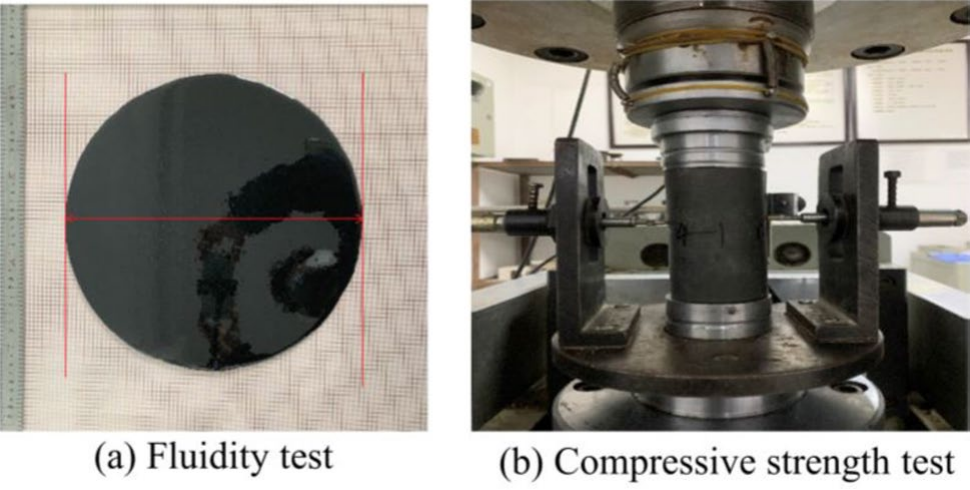
Material performance test.

The experiment utilized magnetic powder consisting of particles with a nanoscale size. The anticipated ratio between the volumes of epoxy resin A liquid and epoxy resin B liquid is 2:1. Each test group consisted of 6% active diluent combined with different ratios of 25%, 40%, and 55% Fe_3_O_4_ powder and flyash. Equations (1) and (2) represent the ratio between Fe_3_O_4_ powder and flyash. Table 1 presents the distribution of each of the nine experimental groups that were created. The test assessed the viscosity, time it took for the material to initially set, time it took for the material to finally set, and the compressive strength of each group, as depicted in Fig. 2.

1 $$\lambda_{1} = \frac{{m_{3} }}{{m_{1} + m_{2} }} \times 100\%$$

2 $$\lambda_{2} = \frac{{m_{4} }}{{m_{1} + m_{2} }} \times 100\%$$

**Table 1:** Test proportioning parameters.

SSerial number	Epoxy resin A/g	Curing agent B/g	Fe_3_O_4_ powder/%	Flyash/%	diluent/%
1	100	50	25	25	6
2	100	50	25	40	6
3	100	50	25	55	6
4	100	50	40	25	6
5	100	50	40	40	6
6	100	50	40	55	6
7	100	50	55	25	6
8	100	50	55	40	6
9	100	50	55	55	6

Among them: $\lambda_{1} ,\,\lambda_{2}$ are the proportion of magnetic powder and flyash ;* m*_*1*_ is the amount of epoxy resin A liquid ; *m*_*2*_ is the amount of epoxy resin B liquid ; *m*_*3*_ is the amount of Fe_3_O_4_ powder ; *m*_*4*_ is the amount of flyash.

### Test results

Based on the above proportional experiments, the experimental data are shown in Table 2.

**Table 2:** Test data statistics.

Serial number	Fluidity/mm	Initial setting time/min	Final setting time/min	Compressive strength/Mpa
				7d
1	446	49	64	57.84
2	439	52	70	63.31
3	412	62	83	67.11
4	405	70	71	47.39
5	423	56	74	54.43
6	441	51	92	59.15
7	448	60	83	44.39
8	437	72	94	52.18
9	397	59	82	57.18

**Figure 3:**
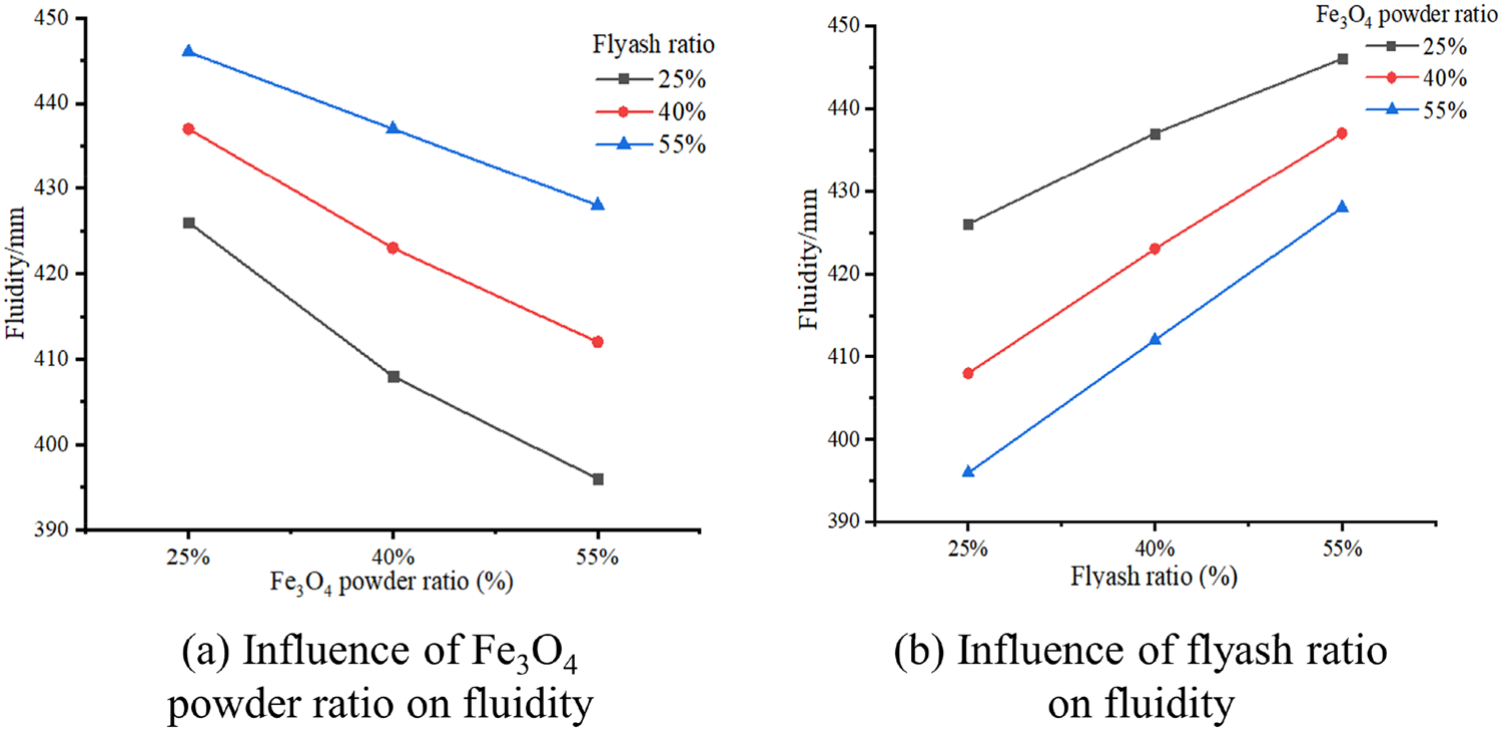
Fluidity change of OMES.

The correlation between the Fe_3_O_4_ particle ratio and fluidity is continuously negative, as seen in Fig. 3a. This correlation is also observed with the fraction of flyash. More precisely, when the ratio increases, the viscosity of the slurry falls. As the amount of fine powder increases and the size of the particles drops, its effect on the angle of repose will become stronger, leading to a loss in fluidity. This is because introducing powder with smaller particles reduces fluidity. Based on the observation in Fig. 3b, it is evident that the flow performance improves when the flyash concentration increases, while keeping the Fe_3_O_4_ powder content constant. The particle size of flyash is bigger than that of Fe_3_O_4_ powder, which is the reason for this difference. The powder with a greater particle size exhibits superior fluidity. To enhance the flow characteristics of a fine powder with limited fluidity, one can introduce larger particles, which will mitigate its tendency to stick together and enhance its ability to flow smoothly. When the blending ratio is between 40 and 55%, the fluidity of the slurry increases at a higher rate compared to a blending ratio of 25%. However, when the blending ratio is 25%, the fluidity of the slurry is the highest.

**Figure 4:**
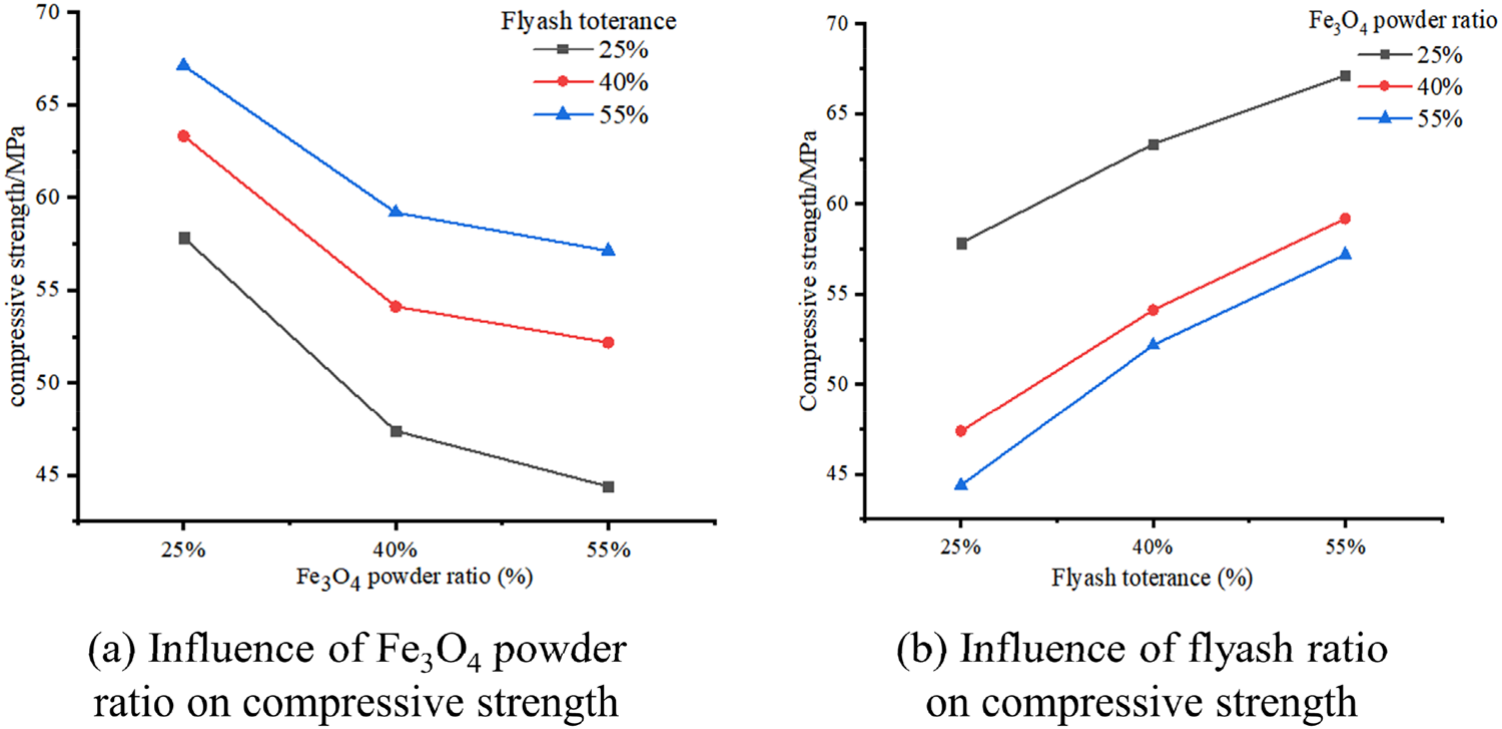
Compressive change of OMES.

The influence of the flyash ratio in the slurry on the consolidation compressive strength is negligible, as shown in Fig. 4a. More precisely, as the amount of Fe_3_O_4_ powder is increased, the compressive strength of the consolidation drops. Moreover, a slight decline in strength was noted in flyash with a mass ratio of 55% and 40%, whereas a significant decline in strength was recorded in flyash with a mass ratio of 25%. The compressive strength of Fe_3_O_4_ powder decreases by 18.1%, 14.5%, and 11.8% when the flyash content is 25%, 40%, and 55%, respectively, in comparison to a 25% flyash concentration. In addition, the compressive strength of Fe_3_O_4_ powder decreases by 23.2%, 17.5%, and 14.8% when the flyash concentration is reduced to 25%.The correlation between the proportion of flyash and consolidation compressive strength is positive, as depicted in Fig. 4b. More precisely, the compressive strength during consolidation falls as the amount of Fe_3_O_4_ powder increases, whereas the proportion of flyash changes. The highest compressive strength is achieved when the content ratio of Fe_3_O_4_ powder is 25%. Additionally, the rate of increase in compressive strength is slightly slower when using 25% and 55% Fe_3_O_4_ powder.

An indoor ratio test was conducted to assess the fundamental attributes of fluidity, setting time, and strength of OMES. The ideal proportion of epoxy resin The ratio between liquid A and liquid B was determined to be 2:1, taking into account the influence of flyash, Fe_3_O_4_ powder admixture, and other parameters on the performance of the slurry. By examining the influence of the ratio of flyash and Fe_3_O_4_ powder on the fundamental characteristics of the slurry, such as its fluidity, setting time, and compressive strength, it has been shown that the ideal mixing ratio falls between the range of 25 and 55%. This discovery is essential for directing the grouting therapy of karst pipeline water seepage.

## Grouting plugging test

### Test system

**Figure 5:**
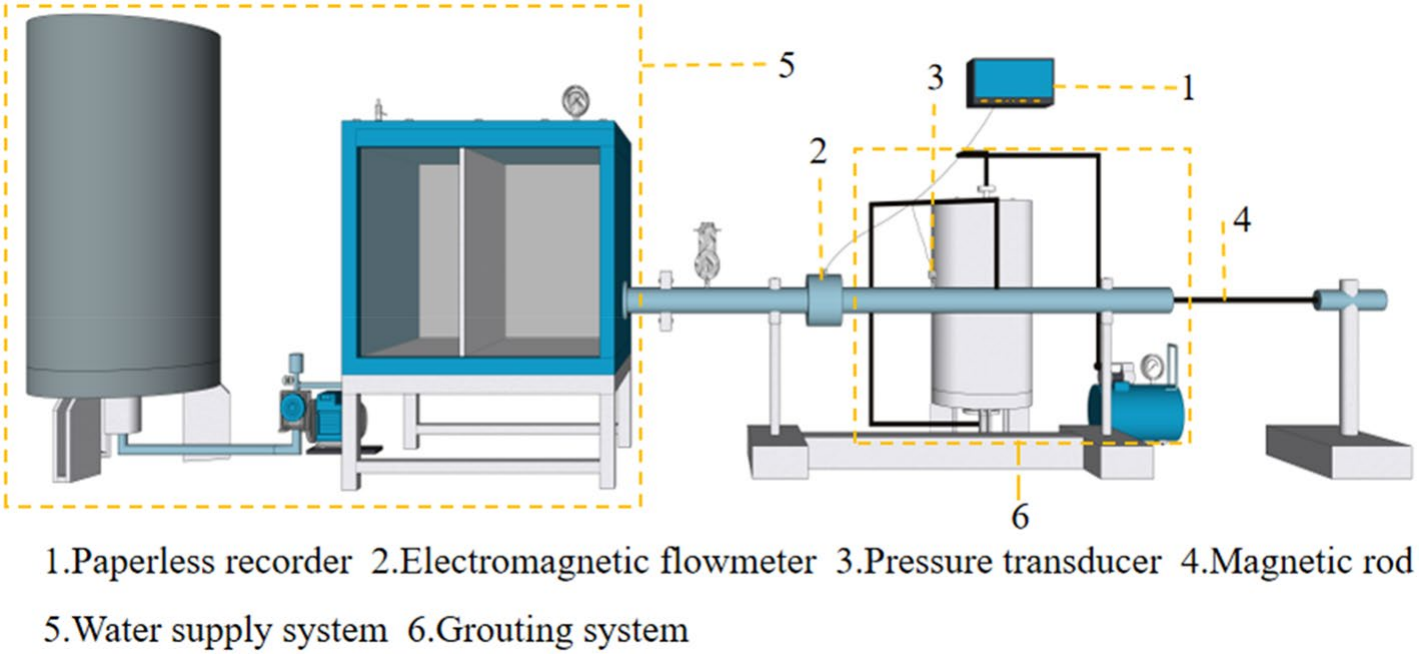
Self-developed grouting plugging simulation experment system.

**Figure 6:**
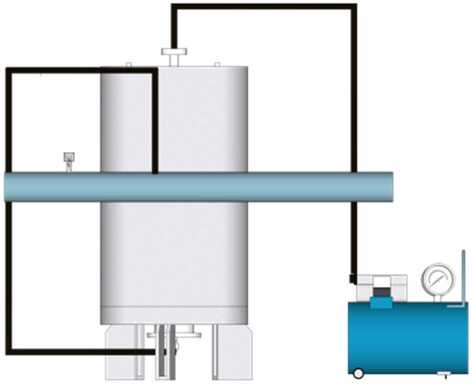
Schematic diagram of the structure of the grouting system.

The karst pipeline's visual grouting plugging simulation test system mentioned in this paper was autonomously developed by China Three Gorges University. The test system is comprised of six components: a steady pressure water supply system, a water pipeline system, a flow control system, a multi-information monitoring system, and a magnetic field system, as seen in Fig. 5. The grouting system, depicted in Fig. 6, was utilized to conduct a series of tests. These studies aimed to determine the impact of dynamic water flow rate, flyash blending ratio, Fe_3_O_4_ powder blending ratio, and plugging length on the effectiveness of plugging. The fundamental principle of pipeline grouting plugging and the ideal flow rate for plugging are determined, offering valuable guidance and reference for on-site grouting.

**Figure 7:**
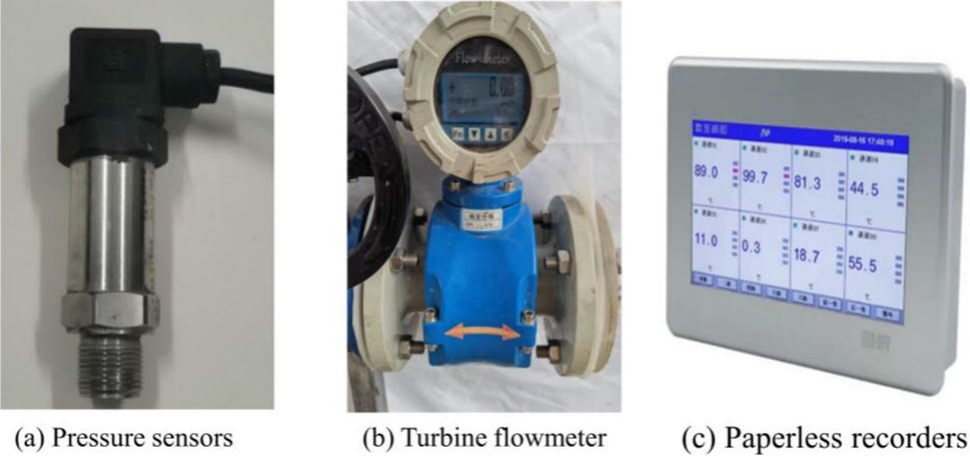
Multi-information monitoring instrument.

During the test, the flow velocity of the karst pipeline gushing water is controlled by the combined action of a water pump and a butterfly valve, taking into account the characteristics of high pressure, huge flow, and high speed. The information monitoring system converts the data into electrical signals using an electromagnetic flowmeter and a pressure transmitter. These signals are then processed by a paperless recorder, as depicted in Fig. 7. The real-time monitoring of the fluid dynamics in the pipeline is conducted. The primary parameters being monitored are water pressure and water flow rate. These measurements are recorded in a paperless recorder with a recording interval of 1 s. Table 3 displays the component parameters of the information monitoring equipment.

**Table 3:** Monitoring components parameters.

Monitoring component	Model	Power supply	Output signal	Measuring medium	Precision (%)	Range
Pressure sersor	HX100	24 VDC	4–20 mA	Liquid	0.5	0–30 m^3^/h
Paperless recorder	MIK-R 9600	220 V	–	–	0.5	0–1000 kpa
Electromagnetic flowmeter	DN50	24 VDC	4–20 mA	Water, oil, slurry	0.5	0–1000 kpa
Electric butterfly valve	D971X-16	220 V	4–20 mA	Water, oil, slurry	0.5	2–40 m^3^/h

### Test scheme

The magnetic slurry filling material investigated in this study demonstrates non-Newtonian fluid characteristics and exhibits reverse thixotropy, wherein its viscosity increases and fluidity decreases with increasing magnetic field strength. An experimental study was carried out to examine the process of grouting and plugging using a magnetic rod with a magnetic flux of 14,000 GS and a diameter of 30 mm. The rod was inserted at the outlet end of a flowing water pipeline system, following the concept of this idea.

The study focused on the visual grouting plugging simulation test device and used the orthogonal experimental design method to investigate the impact of dynamic water flow rate, Fe_3_O_4_ powder blending ratio, flyash blending ratio, and plugging length. The evaluation indexes for the plugging effect of the magnetic slurry pipeline were selected as the slurry retention rate, flow rate change rate, and water plugging pressure in the pipeline. Table 4 displays the factor level table, whereas Table 5 presents the theoretical design of the orthogonal experiment.

**Table 4:** Factor level table.

Factor level	Slurry type	Fe_3_O_4_ power ratio (%)	Flyash mixture ratio (%)	Hydrodynamic velocity (m s^−1^)	Plugging length (mm)
1	OEMS	25	25	0.25	200
2		40	40	0.35	300
3		55	55	0.55	400

**Table 5:** Orthogonal experiment scheme design.

Test number	Slurry type	Fe_3_O_4_ power ratio (%)	Flyash mixture ratio (%)	Hydrodynamic velocity (m s^−1^)	Plugging length (mm)
1	OEMS	25	25	0.25	200
2		25	40	0.35	300
3		25	55	0.45	400
4		40	25	0.35	400
5		40	40	0.45	200
6		40	55	0.25	300
7		55	25	0.45	300
8		55	40	0.25	400
9		55	55	0.35	200

The assessment of the effectiveness of grouting plugging in pipelines under dynamic water circumstances reveals that a higher slurry retention rate and flow rate change rate in the pipe correspond to a superior grouting plugging effect. As the water pressure in the pipeline increases, the dynamic water flow rate decreases, resulting in a more pronounced grouting clogging effect.

## Results and analysis

### Analysis of slurry retention rate

The slurry’s retention rate is indicative of the material’s anti-erosion capabilities to some degree. Utilizing the aforementioned experimental data, the intuitive analysis approach is employed to examine the slurry retention rate test index. This analysis allows for the identification of the primary and secondary relationships between each component that influences the test index. Furthermore, it enables the determination of the most optimal test scheme.

By weighing the quality of the slurry in the slurry recovery device at the outlet of the pipeline, the loss of the dynamic underwater slurry *W*_*1*_ is obtained, and the total amount of slurry injection in the pipeline is *W*_*2*_, and the slurry retention rate is calculated as :

3 $$\eta = \frac{{W_{2} - W_{1} }}{{W_{2} }}$$

The steps of intuitive analysis are as follows:

1. The results of the same level of each influencing factor are added as K_1_, K_2_, K_3_.
2. The average value of the above calculation results is divided by the level number, which is denoted as k_1_, k_2_ and k_3_ respectively.
3. According to the formula $R = \max \overline{k}_{i} - \min \overline{k}_{i}$, the range R is calculated, and the importance order of each influencing factor is analyzed by the range, and the trend chart is drawn based on this.

**Figure 8:**
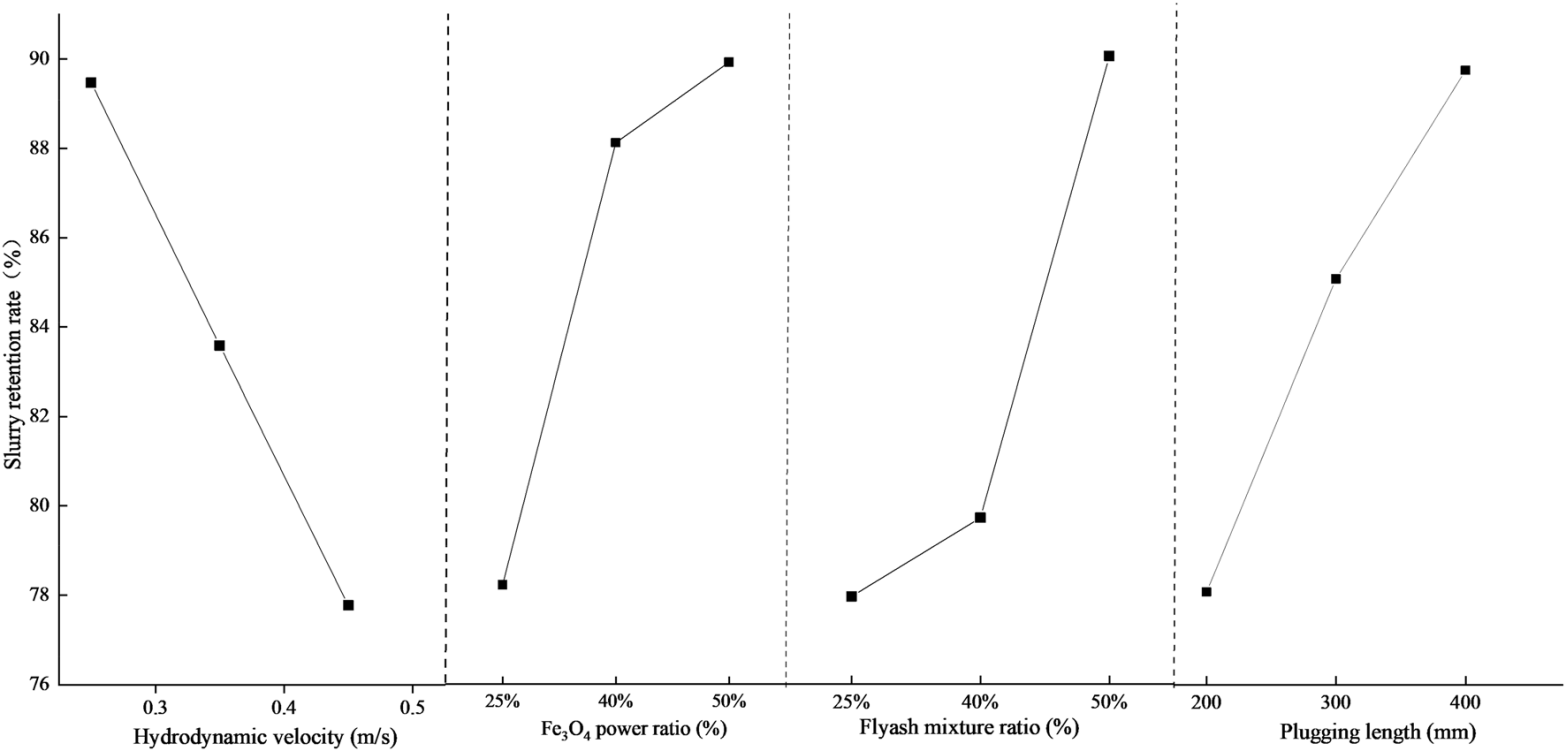
Influre of various factors on slurry retention rate.

Table 6 displays the visual analysis table of the test findings. Based on the aforementioned experimental data, it is evident that the parameters influencing the sealing effect of grouting can be categorized into primary and secondary interactions. The hydrodynamic velocity is greater than the plugging length, which in turn is greater than the Fe_3_O_4_ power ratio, and finally the flyash mixture ratio. Every element examined in Fig. 8 exhibits a substantial link with the slurry retention rate.

1. There is an inverse relationship between the slurry retention rate and the flow velocity. As the water flow rate becomes more dynamic, the hydrodynamic force acting on the slurry in the pipe after grouting will progressively increase. This force surpasses the magnetic field force of the slurry, leading to a stronger erosion effect. Consequently, the slurry retention rate in the pipe decreases.
2. The rate at which the slurry is retained increases as the amounts of Fe_3_O_4_ and flyash increase. A higher concentration of Fe_3_O_4_ and flyash leads to a greater number of magnetic powder particles being completely surrounded by the base fluid. As a result, the slurry has a higher specific gravity and dynamic viscosity, which leads to an enhanced contact force between magnetic particles and an elevation in the internal friction angle. As a result, the magnetic rod attracts and holds more of the slurry, leading to an increase in the slurry retention rate.
3. There is a positive correlation between the slurry retention rate and the plugging length. As the length of the plugs rises, the amount of magnetic slurry adsorbed by the magnetic rod also increases, resulting in a progressive increase in the slurry retention rate in the tube.

**Table 6:** Intuitive analysis table of slurry retention rate.

Facthor	Hydrodynamic velocity (m s^−1^)	Fe_3_O_4_ power ratio	Flyash mixture ratio	Plugging length (mm)
K_1_	268.41	238.46	242.33	238.89
K_2_	250.74	255.22	244.42	255.45
K_3_	233.32	258.81	256.76	266.49
k_1_	89.47	78.48	80.77	79.63
k_2_	83.58	85.07	81.47	85.15
k_3_	77.77	86.27	85.58	88.83
Range	11.70	7.79	4.81	9.20
Primary and secondary factor	Hydrodynamic velocity > Plugging length > Fe_3_O_4_ power ratio > Flyash mixture ratio

### Pressure field and velocity field analysis

The pressure field and velocity field variations in the pipeline are crucial factors for evaluating the effectiveness of grouting plugs. This paper conducts a dynamic water grouting experiment on pipelines, considering various factors such as dynamic water velocity, Fe_3_O_4_ powder mixing ratio, flyash mixing ratio, and plugging length. The study focuses on analyzing the changes in the pressure field and velocity field within the pipeline during the grouting process.

The experimental index is determined by calculating the change rate of flow velocity, which is influenced by the initial flow velocity of moving water in each test. The calculation formula is represented by formula (4). The higher the rate of change in dynamic water velocity within the pipe, the more effective the grouting sealing effect. The range analysis approach is employed to ascertain the impact of the aforementioned elements on the two test parameters: flow rate change rate and water plugging pressure, based on the experimental data. The findings are presented in Table 7 and Table 8.

4 $$v{}_{1} = \frac{{v_{0} - v}}{{v_{0} }} \times 100\%$$

where *v*_*1*_ is the change ratio of the flow velocity; and *v*_*0*_ is the initial flow velocity of the dynamic water; and *v* is the dynamic water velocity after grouting.

**Table 7:** Intuitive analysis table of water plugging pressure.

Facthor	Hydrodynamic velocity (m s^−1^)	Fe_3_O_4_ power ratio	Flyash mixture ratio	Plugging length (mm)
K_1_	58.00	39.00	38.00	30.00
K_2_	39.00	49.00	41.00	45.00
K_3_	27.00	58.00	50.00	54.00
k_1_	19.33	13.00	12.60	10.00
k_2_	13.00	16.33	13.60	15.00
k_3_	9.00	19.33	16.60	18.00
Range	10.33	6.33	3.00	8.00
Primary and secondary factor	Hydrodynamic velocity > Plugging length > Fe_3_O_4_ power ratio > Flyash mixture ratio

**Table 8:** Intuitive analysis table of velocity varition rate.

Facthor	Hydrodynamic velocity (m s^−1^)	Fe_3_O_4_ power ratio	Flyash mixture ratio	Plugging length (mm)
K_1_	159.45	104.93	108.45	109.30
K_2_	112.84	122.92	116.15	117.58
K_3_	96.83	141.27	144.52	148.23
k_1_	53.15	34.97	36.15	34.43
k_2_	37.61	40.97	38.71	39.19
k_3_	32.27	47.09	48.17	49.41
Range	20.88	13	12.02	14.98
Primary and secondary factor	Hydrodynamic velocity > Plugging length > Fe_3_O_4_ power ratio > Flyash mixture ratio			

**Figure 9:**
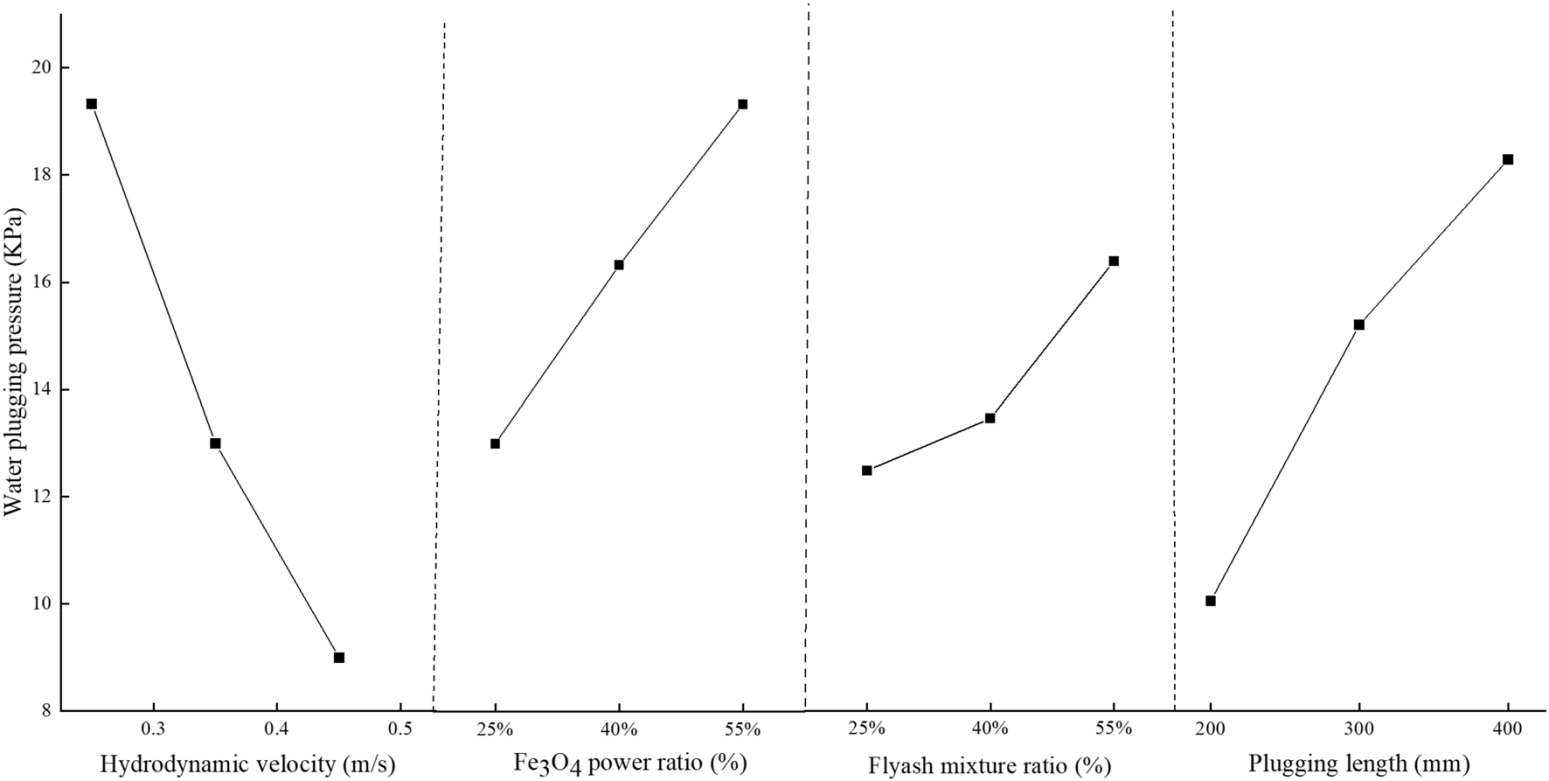
Influre of various factors on water plugging pressure.

**Figure 10:**
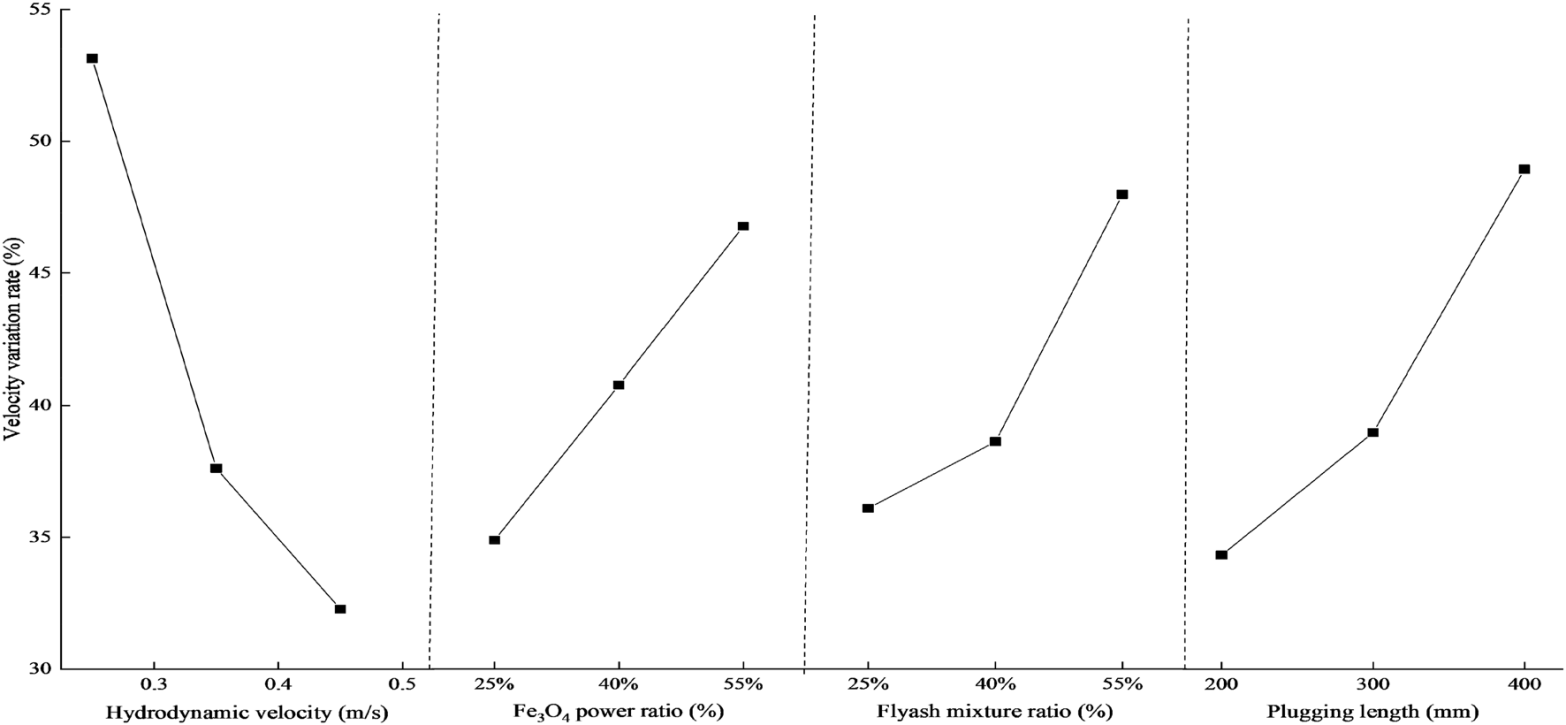
Influre of various factors on water velocity varition rate.

Tables 7 and 8 demonstrate that the flow rate and water plugging pressure in a 50 mm pipe diameter are most influenced by the dynamic water flow rate, followed by Fe_3_O_4_, plugging length, and lastly, the water cement ratio. Figures 9 and 10 demonstrate how several factors affect the rate of velocity change and pressure field.

The following is the trend of elements that influence the rate of flow velocity and water plugging pressure:

1. The rate at which the flow velocity changes and the pressure at which water becomes blocked are inversely related to the dynamic water flow velocity. As the water flow rate becomes higher, the hydrodynamic force acting on the slurry in the pipe after grouting will progressively surpass the magnetic field force of the slurry. The slurry's retention within the pipe will gradually diminish, resulting in a reduction in the water resistance of the slurry. Consequently, the rate of change in both the flow rate and pressure field within the pipe will decrease.
2. There is a direct correlation between the rate at which the flow velocity changes and the water plugging pressure, and the amount of Fe_3_O_4_. As the Fe_3_O_4_ concentration increases, the number of magnetic powder particles fully enveloped by the base fluid also increases. Simultaneously, the slurry experiences an increase in instantaneous viscosity due to magnetization under the influence of a magnetic field. This leads to an increase in the contact force between the magnetic particles and an increase in the internal friction angle. The adhesion of the magnetic rod to the slurry gradually strengthens, hence improving the slurry's water resistance. This not only decreases the flow rate in the tube, but also amplifies the pressure field within the tube.
3. There is a positive correlation between the rate of change of flow velocity and water plugging pressure and the amount of flyash. The quantity of flyash will impact the specific gravity, dynamic viscosity, and rheological characteristics of the slurry. As the amount of flyash increases, the specific gravity and dynamic viscosity of the slurry also increase. This leads to reduced fluidity and increased friction between the slurry and the pipe wall. Consequently, the flow velocity of the water falls and the pressure field in the pipe intensifies.
4. The rate at which velocity changes and the pressure required to plug the water are directly related to the length of the plug. The increase in sealing length leads to a greater amount of magnetic slurry being adsorbed by the magnetic rod. This, in turn, increases the slurry's hindrance to water flow, resulting in a slower speed of dynamic water scouring the slurry. As a result, the rate of slurry loss decreases, leading to a reduction in the dynamic water flow rate and an enhancement of the pressure field in the pipe.

### Analysis of optimal plugging flow rate test

The orthogonal test indicates that the dynamic water flow rate has the most significant impact on pipeline plugging. Furthermore, the higher the flow rate, the more detrimental the grouting plugging effect becomes. Once the dynamic water flow rate beyond a specific threshold, the magnetic slurry loses its ability to adhere to the magnetic rod. The current water flow rate that is constantly changing is referred to as the ideal plugging flow rate. If the dynamic water flow rate in the pipeline exceeds the optimal plugging flow rate, the effectiveness of the grouting operation will be compromised. The slurry retention rate is utilized as the experimental index to measure the effectiveness of varied initial flow velocities of dynamic water in each test. The minimal slurry retention rate in the pipe, which indicates the point at which the magnetic slurry is washed away, is used as the benchmark for achieving the optimal flow rate with the least amount of clogging. In order to streamline the testing process and account for primary and secondary factors that affect the dynamic water flow rate, such as blocking length and flyash amount, a series of exploratory tests were conducted. The initial value of the dynamic water flow rate was set at 0.25 m/s and increased in increments of 0.1 m/s up to 0.75 m/s. A total of 30 test groups were conducted.

**Figure 11:**
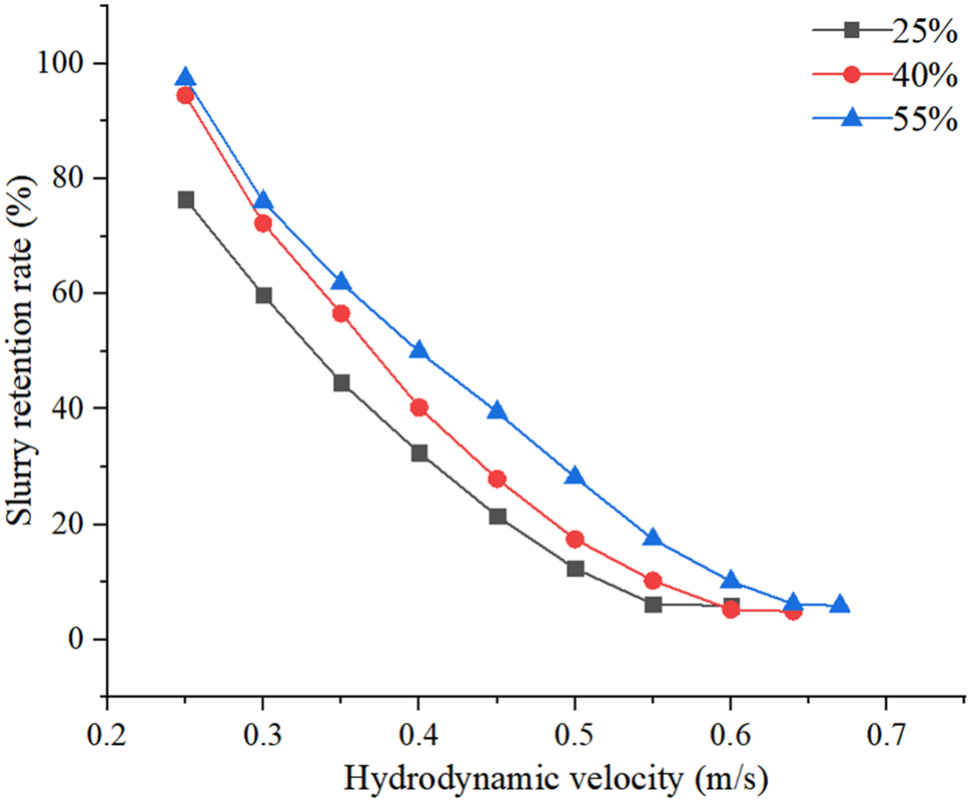
Retention rate of slurry with different Fe_3_O_4_ dosage.

Figure 11 illustrates that there is a significant variation in the slurry retention rate under varied Fe_3_O_4_ when the flow rate of dynamic water is low. The slurry retention rate decreases as the flow rate of dynamic water increases, hence reducing the impact of Fe_3_O_4_. At a low flow rate, the erosive impact of water flow on the slurry is minimal, and the primary determinant of the slurry retention rate is Fe_3_O_4_. As the hydrodynamic velocity increases, the pulsation between water molecules intensifies, and the main factor responsible for this change shifts from Fe_3_O_4_ to the hydrodynamic velocity. The test results indicate that the slurry retention rate is at its lowest when the magnetic slurry contains 25%, 40%, and 55% Fe_3_O_4_ and is flowing at speeds of 0.6 m/s, 0.64 m/s, and 0.67 m/s accordingly. This corresponds to the best plugging flow rate. The ideal flow rate for plugging magnetic slurry varies between 0.6 and 0.67 m/s, depending on the specific working conditions.

## Optimal plugging flow rate model of magnetic slurry

This research assumes the following in order to investigate the optimal plugging flow model of magnetic slurry magnetization:

1. The flow in the water channel is turbulent flow, Reynolds number $R_{e} \ge 4000$;
2. The slurry in the state of water inrush is assumed to be discrete magnetic particle morphology;
3. The magnetic particles have a spherical shape, and the average particle size is considered;
4. The alteration in viscosity of magnetic slurry is not taken into account;
5. Magnetic force is a distinctive penetrating force exhibited by magnetic slurry fluid. The magnetic field exclusively affects Fe_3_O_4_ magnetic particles and does not have any impact on the oily epoxy glue slurry;
6. Magnetic particles and slurry colloids are regarded as a unified entity.

During slurry transit, the magnetic particles within the slurry experience different forces within the water channel, causing them to move and progressively gather and adhere to the magnetic rod. The particle start-up flow rate refers to the minimal flow rate at which magnetic particles become adsorbed around the magnetic rod. When the hydrodynamic velocity surpasses the initial velocity in the given conditions, the magnetic particles are unable to be efficiently adsorbed by the magnetic rod. Currently, the grouting is not effective in sealing the pipeline. Hence, the initial velocity of the magnetic particles is employed as the ideal plugging velocity.

### Force analysis of magnetic particles in flowing water

The force of the magnetic particles in the moving water is very complex. The analysis mainly has the following types:

1. Gravity G.
  5 $${\text{For}}\,{\text{spherical}}\,{\text{particles}}\,W = \frac{{4\pi }}{3}r^{3} \rho _{p} g$$
  In the formula: *r* is the radius of spherical particles, unit *m*, and the equivalent particle size should be considered for non-spherical particles; $\rho_{p}$ is the density of magnetic particles, unit kg/m^3^.
2. Water drag force F_D_^45^
  6 $$F_{D} = \frac{1}{{2}}C_{D} \pi r_{{}}^{2} \rho u^{2}$$
3. Lifting force on water flow F_L_^45^
  7 $$F_{L} = \frac{1}{{2}}C_{L} \pi r^{2} \rho u^{2}$$
4. Magnetic force F_e_ and inter-particle magnetic force F_m_.
  In order to quantitatively describe the mechanical model of magnetic slurry plugging, the magnetic field of a section of the magnetic rod is set as a cylindrical permanent magnet magnetic field, and the magnetic fields between the other sections are not superimposed on each other.

**Figure 12:**
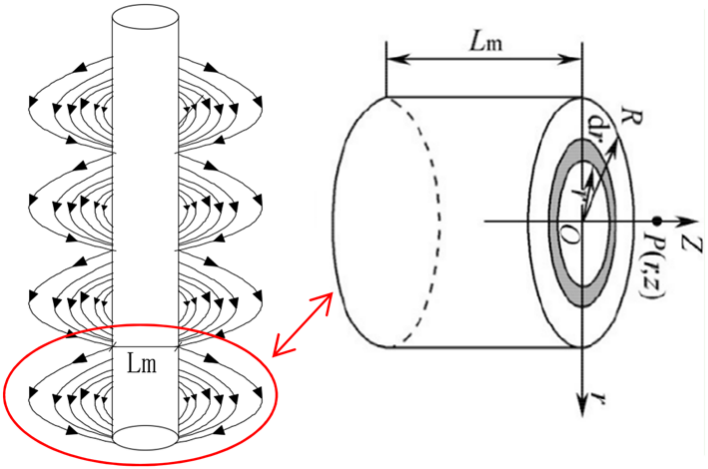
Magnetic field model.

Suppose that the upper pole surface of the lower cylindrical permanent magnet is *N* pole, and its magnetic charge surface density is *Br*, the lower pole surface is *S* pole, and its magnetic charge surface density is *Br*, as shown in Fig. 12. According to the equivalent magnetic charge method, the magnetic induction intensity generated by the magnetic charge of the upper and lower polar surfaces at the *P* point of the axis is:

8 $$B_{PZ1} = \int_{0}^{R} {\frac{{B_{r} }}{4\pi }} \frac{2\pi r}{{(z^{2} + r^{2} )^{{3{/2}}} }}dr = \frac{{B_{r} }}{2}\left( {1 - \frac{z}{{\sqrt {z^{2} + R^{2} } }}} \right)$$

9 $$B_{pz2} = - \frac{{B_{r} }}{2}\left( {1 - \frac{{z + L_{{\text{m}}} }}{{\sqrt {(z + L_{m} )^{2} } + R^{2} }}} \right)$$

Then assume that the magnetic induction intensity *B*_*Pz*_ of point *P* is:

10 $$B_{pz} = \frac{{B_{r} }}{2}(\frac{{z + L_{m} }}{{\sqrt {(z + L_{m} ) + R^{2} } }} - \frac{z}{{\sqrt {z^{2} + R^{2} } }})$$

Then the magnetic field force:

11 $$F_{e} = m\frac{\chi }{{M_{0} }}B = \rho_{p} V\frac{\chi }{{M_{0} }}B_{pz} = \frac{{4\pi r^{3} \rho_{p} \chi B_{pz} }}{{3M_{0} }}$$

Interparticle magnetic force^44^:

12 $$F_{m} = \sum {\left[ {\frac{{3\mu_{0} }}{{4\pi r_{ij}^{5} }}(\vec{m}_{i} \vec{m}_{j} - 5m_{ir} m_{jr} )\vec{r}_{ij} + \frac{{3\mu_{0} }}{{4\pi r_{ij}^{4} }}(m_{ir} \vec{m}_{j} + m_{jr} \vec{m}_{i} )} \right]}$$

In the formula : *r*_*ij*_ is the distance between the center of magnetic particles i and j ;$\vec{m}_{i}$ and $\vec{m}_{j}$ are the magnetic moment vectors of magnetic particles i and j ; *m*_*ir*_ and *m*_*jr*_ are the components of $\vec{m}_{i}$ and $\vec{m}_{j}$ along the $\vec{r}_{ij}$ vector direction of the magnetic particle position ;$\vec{r}_{ij}$ is the position vector from magnetic particle *i* to magnetic particle *j* ; $\mu {}_{{0}}$ is the vacuum permeability ; M_0_ is the magnetization of magnetic particles ; $\chi$ is magnetic susceptibility.

### Single magnetic particle start-up conditions

**Figure 13:**
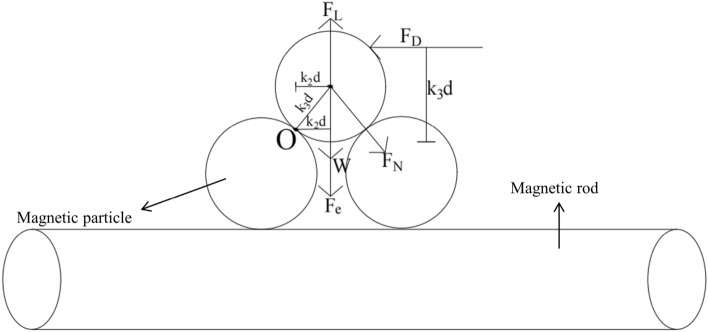
Magnetic particle force analysis diagram.

The force of the magnetic particles in the water channel is shown in Fig. 13. Assuming that the *O* point is the rotation center and ∑M = 0, the critical equation of the magnetic particle starting is established:

13 $${\text{F}}_{{\text{D}}} {\text{K}}_{{3}} {\text{d}} + {\text{F}}_{{\text{L}}} {\text{K}}_{{2}} {\text{d}} = \left( {{\text{W}} + {\text{F}}_{{\text{e}}} } \right){\text{K}}_{{1}} {\text{d}} + {\text{F}}_{{\text{m}}} {\text{K}}_{{4}} {\text{d}}$$

In the formula: K_1d_, K_2d_, K_3d_ and K_4d_ are the corresponding force arms of W + Fe, F_L_, F_D_ and F_m_ respectively. In order to simplify the calculation, K_1_ = K_2_ = K_3_ = K_4_ = 1.

Bring F_D_, F_L_, W, F_e_, F_m_ into Eq. (13), and the critical velocity *u*_*0*_ is:

14 $$u_{0} = \left[ {\frac{1}{{\rho (C_{L} + C_{D} )}}} \right]^{\frac{1}{2}} \left[ {\frac{{8\pi r\rho_{p} (g + \frac{{\chi B_{pz} }}{{M_{0} }})}}{{3\rho (C_{L} + C_{D} )}} + \frac{{F_{m} }}{{\pi r^{2} }}} \right]^{\frac{1}{2}}$$

In the formula : C_D_ and C_L_ are drag coefficient and lift coefficient respectively, taking C_D_ = 0.44, C_L_ = 0.12; the magnetic particle radius r = 5μm, the distance between particles △r = 1.5μm, the vacuum permeability μ_0_ = 4π × 10^−7^ H/m, and the mass susceptibility χ = 59.05 m^3^/kg.

### Probability of starting velocity

Once the water flow velocity reaches the initial velocity of the magnetic particles, the particles within the pipeline will begin to move. Nevertheless, because of the unpredictable arrangement and form of the magnetic particles, there is no specific flow velocity requirement that may cause all the magnetic particles to travel simultaneously. Thus, the probability of the motion of the magnetic particles is regarded as the requirement for the initiation of the magnetic particles.

The probability of magnetic particle starting is:

15 $$\varepsilon = P(u_{1}^{2} \ge u_{0}^{2} )$$

The instantaneous velocity *u*_*1*_ of the magnetic particle starting approximately obeys the normal distribution. Since the probability of the magnetic particle moving in the countercurrent direction is very small, the second term of $\Phi ( \cdot )$ can be ignored^46^. Then Eq. (15) can be expressed as :

16 $$\varepsilon = 1 - \Phi \left( {\frac{{u_{0} - \overline{u}_{1} }}{{\sigma_{1} }}} \right)$$

In the formula: $\Phi ( \cdot )$ is a normal distribution function; $\overline{u}_{1}$ is the uniform velocity of water flow; $\sigma_{{1}}$ is the mean square error of water flow.

In this paper, the influence of the size and position of the magnetic particles on the starting velocity is not considered, and the starting probability corresponding to the general motion is used as the starting probability of the cement particles^47^:

17 $$\overline{u}_{1} = \frac{{u_{0} }}{1.37} = 0.730\,u_{0}$$

Substituting the formula (17) into (14), the starting flow velocity after considering the probability of flow velocity is :

18 $$\overline{u}_{1} = 0.730\left[ {\frac{1}{{\rho (C_{L} + C_{D} )}}} \right]^{\frac{1}{2}} \left[ {\frac{{8\pi r\rho_{p} (g + \frac{{\chi B_{pz} }}{{M_{0} }})}}{{3\rho (C_{L} + C_{D} )}} + \frac{{F_{m} }}{{\pi r^{2} }}} \right]^{\frac{1}{2}}$$

### Criteria for determining optimal sealing flow rate

**Figure 14:**
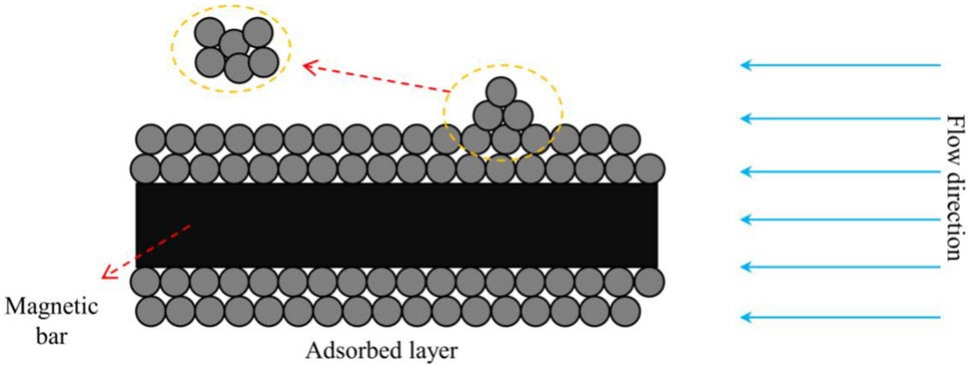
The starting diagram of magnetic particle cluster.

The motion of magnetic particles in the water inflow pipeline is not individual, but typically occurs in a collective manner, as depicted in Fig. 14. By employing principles from fluid mechanics, magnetic fluid mechanics, and particle sedimentation theory, the analysis focuses on the force exerted by magnetic particle units. Through adjusting the initial velocity of magnetic particles, the optimal flow velocity for magnetic particle agglomeration is determined.

The optimal plugging flow rate formula of magnetic slurry is:

19 $$\overline{u}_{2} = 0.730\left[ {\frac{1}{{\rho (C_{L} + C_{D} )}}} \right]^{\frac{1}{2}} \left[ {\frac{{8\pi r\rho_{p} (g + \frac{{\chi B_{pz} }}{{M_{0} }})}}{{3\rho (C_{L} + C_{D} )}} + \frac{{F_{m} }}{{\pi r^{2} }}} \right]^{\frac{1}{2}}$$

To summarize, the magnetic particle group's flow rate is utilized as the ideal flow rate for plugging the slurry. Therefore, the criterion for determining the optimal plugging flow rate is as follows: when the time-averaged flow rate is less than the optimal plugging flow rate, the magnetic particles can effectively adsorb and agglomerate around the magnetic rod under dynamic water conditions, effectively blocking the water inflow channel.

### Verification of theoretical model and experimental results

Based on the aforementioned studies, it can be inferred that the ideal velocities for plugging with various ratios of magnetic slurry are 0.6 m/s, 0.64 m/s, and 0.67 m/s, respectively. The optimal plugging velocity model incorporates the different operating conditions and material factors of the test. Theoretical values for the speeds are 0.55 m/s, 0.58 m/s, and 0.6 m/s, respectively. The relative inaccuracy is negligible, and the theoretical calculation values are lower than the indoor test results. The primary factors contributing to the discrepancy between the theoretical and experimental values are as follows: ① The theoretical model is predicated on specific assumptions that do not align entirely with the real-world circumstances. ② Human mistake resulting from deviations in test procedures, such as inadequate reading or lack of expertise in using instruments. The similarity between the experimental value and the theoretical value, along with the tiny margin of error, confirms the validity of the optimal sealing flow rate model.

## Conclusion

This research investigates the diffusion and plugging mechanism of grouting slurry in the presence of water influx through a combination of laboratory testing and theoretical analysis. Initially, a novel form of OEMS was created. The study focused on investigating the diffusion and plugging process of magnetic slurry in a self-developed visual water inrush grouting simulation test system. Subsequently, a theoretical derivation was used to create the migration model of magnetic slurry in a magnetic field when water inrush occurs. The accuracy of the theoretical model is confirmed by comparing the theoretical outcomes with the experimental outcomes. The findings of this investigation are condensed as follows:

1. By analyzing the fundamental properties of OEMS, including fluidity, setting time, and strength, it has been established that the optimal ratio of grouting material in epoxy resin A liquid and B liquid is 2:1. Additionally, the optimal blending ratio of flyash and Fe_3_O_4_ powder for achieving the best grouting sealing performance ranges from 25 to 55%.
2. The orthogonal test was conducted to evaluate the effectiveness of the magnetic self-polymerizing slurry sealing method under dynamic water flow conditions. The evaluation indexes for this study were the water plugging pressure, flow rate change rate, and slurry retention rate. The primary and secondary relationships of the influencing factors for each evaluation index were determined using range analysis.
3. The orthogonal experiment was used to determine the primary and secondary association between each element and the assessment index. The hydrodynamic velocity is more than the plugging length, which in turn is greater than the Fe_3_O_4_ power ratio, and finally, the flyash mixture ratio. There is a positive correlation between the length of water plugging and the amount of Fe_3_O_4_ powder, and the slurry retention rate and water plugging pressure rise as these factors increase.
4. A migration model is developed to study the movement of magnetic slurry in a magnetic field when water influx occurs. The critical flow rate at which the magnetic particles initiate movement is considered as the criterion for determining the appropriate plugging flow rate. The model offers theoretical guidance for the magnetic slurry grouting plugging process, taking into account several elements that influence the appropriate flow rate for plugging under different situations.
5. The findings from the indoor test on the best flow rate for plugging indicate that the ideal flow rate for magnetic slurry varies between 0.6 and 0.67 m/s, depending on the specific operating conditions. The computed value of the theoretical model aligns closely with the experimental value, and the relative error is minimal, confirming the validity of the theoretical model.

## Data Availability

The datasets used and/or analyzed during the current study are available from the corresponding author on reasonable request.
